# Seclusion Reviews: Creation of a Training Video by and for Resident Doctors

**DOI:** 10.1192/bjo.2026.11471

**Published:** 2026-06-30

**Authors:** Luke Robinson, Promita Saha, Caroline Brewer, Awele Onwuli, Emma McPhail

**Affiliations:** 1 Derbyshire Healthcare NHS Foundation Trust, Derby, United Kingdom; 2 Nottinghamshire Healthcare NHS Foundation Trust, Nottingham, United Kingdom

## Abstract

**Aims:**

To improve induction to medical seclusion reviews (MSRs) for Resident Doctors (RDs) rotating into psychiatry in Derbyshire Healthcare NHS Foundation Trust (DHCFT).

Concerns were previously raised by rotating RDs in DHCFT regarding induction to seclusion and feeling unprepared for leading MSRs. Seclusion, as restrictive practice with potential large impacts on our patients, is closely audited both nationally and locally as per the Mental Health Act Code of Practice. Many RDs are not psychiatry trainees and are therefore unfamiliar with the process. Nursing staff within the trust had also reported concerns regarding safety awareness of RDs during MSRs particularly soon after newly rotating into the trust.

**Methods:**

We developed a video of a mock scenario encompassing all aspects of a MSR including timing and structure of reviews based on both local guidelines and the Mental Health Act Code of Practice. This video was recorded within a seclusion suite to demonstrate the layout. This was shown to current RDs 3 months into their rotation. A survey wascompleted on previous experience of seclusion, confidence of completing seclusion reviews, whether had been previously inducted on seclusion and preparedness to complete MSRs prior to viewing the video. Results were compared with a follow-up survey after viewing the video assessing its impact.

**Results:**

Of the 29 respondents to the initial survey; 79% (n=23) reported having no previous induction to seclusion. Respondents were a mixture of Foundation Doctors, GP trainees, Psychiatric Core trainees and Psychiatric Higher Specialty trainees. Mean confidence completing seclusion reviews was 2.66/5. 93% (n=27) of respondents stated they were not prepared enough to complete a MSR after induction. 22 reponses were received to the follow-up survey. Mean confidence completing seclusion reviews increased to 4.23/5. 100% of respondents stated a video on seclusion in induction would be helpful.

**Conclusion:**

This video has demonstrated a novel, relevant and sustainable approach to delivering induction for RDs surrounding a unique aspect of psychiatry which had previously been insufficient in preparing newly rotating RDs for completing seclusion reviews. This video is being further shared with East Midlands School of Psychiatry so that it can contribute to shared learning across other trusts within the region.

